# Serum levels of IL-6 are associated with cognitive impairment in the salus in apulia population-based study

**DOI:** 10.1016/j.heliyon.2023.e13972

**Published:** 2023-02-22

**Authors:** Chiara Griseta, Petronilla Battista, Fabio Castellana, Isabella Colonna, Sabrina Sciarra, Roberta Zupo, Ilaria Bortone, Luisa Lampignano, Sarah Tirelli, Giuseppe Bernardino, Anita Mollica, Madia Lozupone, Francesco Panza, Pietro Fiore, Brigida Minafra, Rodolfo Sardone

**Affiliations:** a Istituti Clinici Scientifici Maugeri IRCCS, Laboratory of Neuropsychology, Bari Institute, Italy; b National Institute of Gastroenterology “Saverio de Bellis,” Research Hospital, Castellana Grotte, Bari, Italy; c Istituti Clinici Scientifici Maugeri IRCCS, Bari Institute, Italy

**Keywords:** Aging, Cognition, Inflammation, Chronic disease, Rehabilitation, *C*-reactive protein, Tumor necrosis factor-alpha

## Abstract

Growing evidence suggests that inflammation contributes to brain aging and neurodegeneration. This study investigates the relationship between global cognitive as well executive function and the inflammatory markers IL-6, CRP, and TNF-α in a population-based study of older adults. A population-based sample, of older people in Southern Italy, was enrolled. We measured serum levels of IL-6, CRP, and TNF-α. We also administered two neuropsychological tests: Mini-Mental State Examination and Frontal Assessment Battery. Rank-based regression models were performed to investigate the relationship between inflammatory markers and cognitive functions, including major demographic and clinical confounders for adjustment. The sample consisted of 1929 subjects aged between 65 and 95 years. Multivariate linear regression analysis revealed that higher serum levels of IL-6 were associated with lower MMSE and FAB scores even after adjustment for demographic data and cardiovascular risk factors. No significant associations were found between cognitive functioning and serum levels of CRP and TNF-α. Our results suggest that higher levels of IL-6 were associated with cognitive impairment in an older adult population of Southern Italy.

## 1 Introduction

Chronic low-grade inflammation is one of the main physiological processes that occur with aging, characterized by high levels of inflammatory markers in older adults. In the context of age-related brain changes, peripheral low-grade inflammation has been shown to contribute to the physiological and pathological decline of cognitive functions and to the etiology of dementia [1,2]. Several studies found inflammation to be a relevant mechanism underlying Alzheimer's disease (AD) pathophysiology, besides the accumulation of amyloid plaques and neurofibrillary tangles [3]. The pathophysiological mechanisms underlying inflammation involve the activation of microglia cells, responsible for the release of several cytokines and toxic products, including reactive oxygen species and nitric oxide. Among the cytokines implicated in the inflammatory processes, interleukins (IL) and tumor necrosis factors (TNF) are particularly relevant in promoting the immune reaction. IL-6 is among those cytokines released by the immune system after an infection or tissue injury stimulation. When IL-6 is secreted, it moves through the bloodstream, stimulating the production of proteins in the liver, like the *C*-reactive protein (CRP), in the acute phase of the inflammatory process. The acute inflammatory response is also responsible for the production of TNF-α, a pro-inflammatory cytokine released by macrophages and monocytes whose activation contributes to a cascade of events within cells, causing necrosis or apoptosis [4]. Cytokine dysregulation in the brain might contribute to cognitive decline via synaptic regression and apoptosis of cells, dysregulation of neurotransmitters, demyelination, or neuronal death (Schram et al., 2007).

The identification of cytokines involved in cognitive decline might play an important role in the development of new potential therapeutic agents. However, the role of peripheral low-grade inflammation in age-related processes and cognitive decline is still to be examined to disentangle the inconsistency coming from differences in the study design of the previous works. Further, it has been shown that the effects of inflammation might be different depending on ethnic characteristics [5] and socio-economic disparities [6]. So far, no previous study explored the association between cognition and neuroinflammation in older adults of South Italy, which is a middle-income macroregion of Italy consisting of a population with a high proportion of older adults (24% aged 60 years and over). Therefore, the aim of this community-dwelling study was to investigate the association between the variation of the serum levels of inflammatory biomarkers (IL-6, TNF-α, CRP) and quantitative measures of global cognitive function (MMSE) and executive function (FAB) in South-Italian older adults.

## 2 Methods

### 2.1 Study population

We obtained informed consent from all participants involved in a population-based study on aging named the “Salus in Apulia Study”, an ongoing population-based prospective cohort study comprising 2472 aged over 65, recruited from the electoral rolls of Castellana Grotte, a town near Bari, Puglia, in Southeast Italy. Further details about the methodology of the study have been reported in previous studies (i.e., [7]. The aim of the Salus in Apulia population-based study is to investigate the impact of nutrition and age-related hearing loss as predictors of neurodegenerative diseases and frailty phenotypes in older adults (i.e., [8,9]. The “Salus in Apulia Study” is supported by the Italian 10.13039/100009647Ministry of Health and Apulia Regional Government as a health-related program conducted by 10.13039/501100014835IRCCS “Saverio De Bellis” Research Hospital. Data from two previous populations are combined within the Salus in Apulia population-based study: a baseline (MICOL3, M3: 2003–2005) and a follow-up cohort (GreatAGE Study - MICOL4, M4: 2013–2015). All procedures performed in the study were in accordance with the Helsinki Declaration. The local IRB of the head institution, IRCCS “Saverio de Bellis” in Castellana Grotte (Apulia, Southern Italy), approved the study. “Standards for Reporting Diagnostic Accuracy Studies” (STARD) guidelines (http://www.stard-statement.org/) have been followed in this study, together with the “Strengthening the Reporting of Observational Studies in Epidemiology” (STROBE) standards.

### 2.2 Cognitive evaluation

Global cognitive function was assessed with the Mini-Mental State Examination (MMSE) [10,11]. In the clinical practice, MMSE is used in order to detect cognitive impairment, to monitor cognitive decline over time, and to evaluate the impact of potential treatments on cognition. It has the advantage to be short and easy to administer and to score. The MMSE includes ten items assessing multiple cognitive functions, including attention, orientation, and calculation, language (comprehension and production), and immediate and delayed verbal memory. The maximum MMSE score is 30, with scores ≤24 points denoting the presence of cognitive impairment. Executive functions were evaluated with the Frontal Assessment Battery (FAB) [12]. FAB is a short cognitive-executive battery suitable to the bedside evaluation of both cognitive and motor functions; it includes the evaluation of mental flexibility, the ability to conceptualize, motor programming, resistance to interference, inhibitory control and environmental autonomy. Each subtest has a score ranging from 0 to 3, the total score ranges from 0 to 18, with scores ≤13 denoting an impairment in executive functions. For all included individuals, we collected data about years of education.

### 2.3 Clinical and laboratory examination

Clinical information regarding the presence of depressive symptoms were obtained from self-reported histories, medical records, and semi-structured interviews (SCID-I) [13]. Depression was diagnosed according to the American Psychiatric Association criteria [14].

Anthropometrical parameters were obtained with participants standing barefoot and wearing loose-fitting clothes. Each measurement was evaluated always between 7:00 and 10:00 a.m., following one-night fast. A wall-mounted stadiometer (Seca 711; Seca, Hamburg, Germany) was used to measure height to the nearest 0.5 cm. A calibrated balance beam scale (Seca 711; Seca, Hamburg, Germany) was employed to measure body weight to the nearest 0.1 kg. Body Mass Index (BMI) was calculated by dividing body weight (Kg) by height (m2) and was then categorized in accordance with World Health Organization standards [15]. Blood examinations were performed using standard automated enzymatic colorimetric techniques (AutoMate 2550, Beckmann Coulter, Brea, CA, US) in order to assess fasting blood glucose (FBG), glycated hemoglobin (HbA1c), total cholesterol, high-density lipoprotein (HDL) cholesterol, and low-density lipoprotein (LDL) cholesterol, red blood cells (RBC), white blood cells (WBC), platelets and triglycerides. In order to assess alanine aminotransferase (ALT), aspartate aminotransferase (AST) and gamma-glutamyl transferase (GGT), automatic enzyme techniques were employed. The glucose oxidase method (Sclavus, Siena, Italy) was used for the evaluation of plasma glucose. The levels of insulin in the blood were measured by radioimmunoassay (Behring, Scoppito, Italy). We applied a latex particle-enhanced immunoturbidimetric test (Kamiya Biomedical Company, Seattle, WA) to assay serum high-sensitivity *C*-reactive protein (hs-CRP) (reference range: 0–5.5 mg/L; interassay coefficient of variation: 4.5%). Using the quantitative sandwich enzyme technique of the ELISA (QuantiKine High Sensitivity Kit, R&D Systems, Minneapolis, MN, and QuantiGlo immunoassay from R&D Systems, Minneapolis, MN), IL-6 and TNF-α were quantified. For IL-6 and TNF-α, the inter-assay coefficient of variability was 11.7% and 13.0%, respectively. Strict quality control techniques were performed during assessing inflammatory marker assays at the same laboratory. During the clinical evaluation, we assessed systolic (SBP) and diastolic (DBP) blood pressure employing OMRON M6 automatic sphygmomanometer in a seated position and after a 10 min rest.

### 2.4 Statistical analysis

The Kolmogorov-Smirnov test was used to test normal distributions of quantitative variables. Therefore, data were reported as median (IQR) for continuous variables, and as frequency and percentages for all categorical variables. A nonparametric approach (Spearman's test) was used to assess correlations among collected variables, for quantitative data. A p-value ≤0.05 was considered significant. A corrplot has been performed to visualize all Spearman's correlation matrices.

Four nested rank-based linear models were generated to examine the relationships between the inflammatory markers (i.e., IL-6, CRP, and TNF-α) and each cognitive score (i.e., MMSE and FAB), separately. The first model calculated unadjusted regression coefficients. The second model included analyses adjusting for demographic factors (age, sex, and education). The same analyses were repeated in a third model, additionally accounting for the clinical covariates that we found to be significant in the previous Spearman's correlation test. The clinical variables found to be significant and therefore included in the analysis were: Body Mass Index (BMI), low-density lipoprotein (LDL) cholesterol, aspartate aminotransferase (AST), red blood cells (RBC), and depression. These confounding variables were initially chosen based on literature research and availability, then only those that we found to be significant in the previous correlation matrix were included in analyses in order to avoid multicollinearity in the models. Finally, the fourth model calculated cognitive measures (i.e., MMSE and FAB, separately) and IL-6 serum levels as regressors corrected by Model 3 confounding variables and CRP and TNF-α serum levels. This model was implemented with other cytokines to exclude the modification effect in the association found between IL-6 and cognitive functions. Data analysis was conducted employing R studio 2021.09.1.

## 3 Results

### 3.1 Characteristics of the sample

Of the original cohort, the final sample consisted of n = 1929 subjects aged between 65 and 95 years (median = 72, IQR = 10) of which n = 974 (50.5%) were males. Years of education ranged from 0 to 23 years (median = 5, IQR = 3). All the included subjects underwent the cognitive (i.e., MMSE; FAB) as well as clinical and laboratory evaluation, including serum measures of IL-6, CRP, and TNF-α. Table 1 summarizes the sociodemographic, clinical characteristics, and inflammatory biomarkers of older participants.

**Table 1:** Demographic and clinical characteristics of the whole sample. N:1929 All data are shown as median (IQR) for continuous variables and as n (%).

	Median	IQR
Age (years)	72	10
Sex		
*Male*	974 (50.50)	
*Female*	955 (49.50)	
Educational level (years)	5	3
MMSE	28	4
FAB	14	6
Depression (yes)	226 (13.00)	
IL-6 (pg/ml)	1.93	1.89
TNF-α (pg/ml)	2.18	1.52
CRP (mg/dl)	0.32	0.52
BMI (kg/m^2^)	28.06	6.40
DBP (mmHg)	80	10
SBP (mmHg)	130	20
FBG (mg/dl)	99	20
HB A1c (mmol/mol)	39	10
Insulin (UI)	7.3	6.52
LDL Cholesterol (mg/dl)	112	43
HDL Cholesterol (mg/dl)	46	17
Total Cholesterol (mg/dl)	183	52
Triglycerides (mg/dl)	93	61
RBC (10^6^ cells/mm^3^)	4.77	0.65
WBC (10^3^ cells/mm^3^)	5.90	2.01
Platelets (10^3^ cells/mm^3^)	216	69
AST (U/L)	23	15
ALT (U/L)	19	13
GGT (U/L)	19	21

*Mann Whitney *U* test for continuous variables and Chi Squared test for categorical ones.

Legend: MMSE: Mini-Mental State Examination; FAB: Frontal Assessment Battery; BMI: Body Mass Index; DBP:Diastolic Blood Pressure; SBP: Systolic Blood Pressure; FBG: Fasting Blood Glucose; HB A1c: Glycated Hemoglobin; RBC: Red Blood Cells; WBC: White Blood Cells; AST: Aspartate aminotransferase:ALT:alanine aminotransferase; GGT:Gamma Glutamyl Transferase; IL-6:Interleukin 6; TNF-α: Tumor Necrosis Factor Alpha; CRP: *C*-reactive Protein.

### 3.2 Correlations between markers of inflammation and cognitive performance

**Fig. 1:**
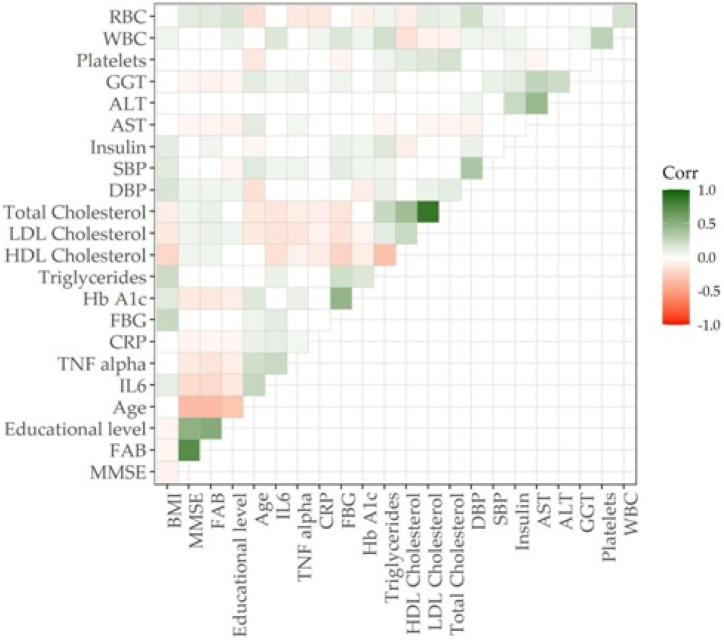
Spearman's correlation plot of MMSE and FAB and collected variables. Legend: BMI: Body Mass Index, MMSE: Mini-Mental Evaluation Scale, FAB: Frontal Assessment Battery, DBP: Diastolic Blood Pressure, SBP: Systolic Blood Pressure, FBG: Fasting Blood Glucose, Hb A1c: Glycated Hemoglobin, RBC: Red Blood Cells, WBC: White Blood Cells, AST: aspartate aminotransferase, ALT: Alanine Transaminase, GGT: gamma-glutamyl transferase, TNF-α: Tumor Necrosis Factor-alpha, CRP: *C*-reactive Protein.

In our cohort, MMSE and FAB scores were inversely associated with age and directly associated with years of education (Fig. 1; Table 2). Further, higher BMI was related to poorer performance on the MMSE (Rho: −0.07; 95% CI: −0.11 to −0.02). HbA1c values were inversely associated with MMSE (Rho: −0.11; 95% CI: −0.15 to −0.07) and FAB (Rho: −0.12; 95% CI: −0.16 to −0.07), while no significant associations were found for FBG. Conversely, higher values of total cholesterol, LDL, and HDL were related to a greater MMSE and FAB scores (Table 2), while such association was not found for triglyceride.

**Table 2:** Spearman's correlation matrix of MMSE and FAB and collected variables.

	MMSE		FAB	
	Rho	CI 95%	Rho	CI 95%
BMI (kg/m^2^)	−0.07	−0.11 to −0.02	−0.06	−0.10 to 0.02
FAB/MMSE	0.73	0.71 to 0.75	0.73	0.71 to 0.75
Education (years)	0.49	0.45 to 0.52	0.53	0.49 to 0.55
Age (years)	−0.36	−0.39 to −0.31	−0.37	−0.4 to −0.32
IL-6 (pg/ml)	−0.19	−0.23 to −0.14	−0.2	−0.23 to −0.15
TNF-α (pg/ml)	−0.11	−0.16 to −0.06	−0.14	−0.17 to −0.09
CRP (mg/dl)	−0.06	−0.1 to −0.01	−0.05	−0.99 to −0.01
FBG (mg/dl)	−0.03	−0.07 to 0.01	−0.03	−0.07 to 0.02
Hb A1c (mmol/mol)	−0.11	−0.15 to −0.07	−0.12	−0.16 to −0.07
Triglycerides (mg/dl)	−0.03	−0.07 to 0.09	0.03	−0.01 to 0.07
HDL Cholesterol (mg/dl)	0.06	0.01 to 0.1	0.07	0.04 to 0.11
LDL Cholesterol (mg/dl)	0.07	0.02 to 0.11	0.08	0.03 to 0.12
Total Cholesterol (mg/dl)	0.06	0.01 to 0.1	0.1	0.05 to 0.13
DBP (mmHg)	0.06	0.01 to 0.1	0.06	0.02 to 0.1
SBP (mmHg)	−0.02	−0.06 to 0.02	−0.04	−0.08 to 0.01
Insulin (UI)	0.02	−0.02 to 0.06	0.06	0.02 to 0.10
AST (U/L)	−0.05	−0.02 to −0.01	−0.06	−0.1 to −0.01
ALT (U/L)	0.01	−0.03 to 0.05	0.03	−0.01 to 0.07
GGT (U/L)	−0.05	−0.08 to 0.01	−0.07	−0.11 to −0.03
Platelets (10^3^ cells/mm^3^)	−0.01	−0.05 to 0.05	0.01	−0.03 to 0.05
WBC (10^3^ cells/mm^3^)	−0.01	−0.05 to 0.02	−0.01	−0.05 to 0.03
RBC (10^6^ cells/mm^3^)	0.12	0.08 to 0.16	0.13	0.08 to 0.17

Legend: BMI: Body Mass Index, MMSE: Mini Mental Evaluation Scale, FAB: Frontal Assessment Battery, DBP: Diastolic Blood Pressure, SBP: Systolic Blood Pressure, FBG: Fasting Blood Glucose, Hb A1c: Glycated Hemoglobin, RBC: Red Blood Cells, WBC: White Blood Cells, AST: aspartate aminotransferase, ALT: Alanine Transaminase, GGT: gamma-glutamyl transferase, TNF-α: Tumor Necrosis Factor alpha, CRP: *C*-reactive Protein.

With regard to liver function, AST levels were inversely correlated with MMSE (Rho: −0.05; 95% CI: −0.02 to −0.01) and FAB (Rho: −0.06; 95% CI: −0.1 to −0.01) scores, while greater GGT values were associated only with lower FAB scores (Rho: −0.07; 95% CI: −0.11 to −0.03), no association was found for ALT. Higher insulin was related to a greater FAB score.

With regard to the inflammatory markers, no significant associations were found between higher WBC and neuropsychological tests, while higher levels of CRP, IL-6, and TNF-α were associated with poorer performance on MMSE and FAB (Table 2).

### 3.3 Markers of inflammation as predictors of cognitive performance

The results of the four ranked-based regression models are reported separately for MMSE and FAB, in Table 3a and Table 3b, respectively.

**Table 3A:** Rank-based Regression model on MMSE as a dependent variable and IL6 serum level as a regressor.

	Coefficient	Stand. Err.	CI 95%
	**Model 1**		
IL-6 (pg/ml)	−0.13	0.01	−0.14 to −0.12
	**Model 2**		
Interleukin 6 (pg/ml)	−0.10	0.01	−0.12 to −0.09
Age (years)	−0.15	0.01	−0.17 to −0.13
Sex (Female)	−0.37	0.13	−0.62 to −0.11
Educational level (years)	0.29	0.02	0.25 to 0.32
	**Model 3**		
IL-6 (pg/ml)	−0.10	0.01	−0.12 to −0.08
Age (years)	−0.15	0.01	−0.17 to −0.13
Sex (Female)	−0.34	0.14	−0.61 to −0.06
Educational level (years)	0.28	0.02	0.25 to 0.32
BMI (kg/m^2^)	−0.03	0.01	−0.06 to −0.01
LDL Cholesterol (mg/dl)	0.01	0.01	−0.02 to 0.03
AST (U/L)	0.01	0.01	−0.02 to 0.03
RBC (10^6^ cells/mm^3^)	0.02	0.07	−0.11 to 0.15
Depression (yes)	−0.54	0.20	−0.94 to −0.15
	**Model 4**		
IL-6 (pg/ml)	−0.10	0.01	−0.12 to −0.08
Age (years)	−0.15	0.01	−0.17 to −0.13
Sex (Female)	−0.35	0.14	−0.63 to -0.08
Educational level (years)	0.28	0.02	0.25 to 0.32
BMI (kg/m^2^)	−0.03	0.01	−0.06 to 0.01
LDL Cholesterol (mg/dl)	−0.01	0.01	−0.02 to 0.03
AST (U/L)	−0.01	0.01	0.02 to 0.03
RBC (10^6^ cells/mm^3^)	0.02	0.07	−0.12 to 0.15
Depression (yes)	−0.53	0.20	−0.93 to −0.14
CRP (mg/dl)	0.02	0.08	−0.13 to 0.18
TNF-α (pg/ml)	0.04	0.02	0.01 to 0.07

Legend: BMI: Body Mass Index, AST:aspartate aminotransferase, RBC: Red Blood Cells, CRP: *C*-reactive Protein, TNF -α: Tumor Necrosis Factor alpha.

**Table 3B:** Rank-based Regression model on FAB as a dependent variable and IL-6 serum level as a regressor.

	Coefficient	Stand. Err.	CI 95%
	Model 1		
IL-6 (pg/ml)	−0.11	0.01	−0.12 to −0.09
	Model 2		
IL-6 (pg/ml)	−0.05	0.01	−0.08 to −0.03
Age (years)	−0.16	0.01	−0.19 to −0.14
Sex (Female)	0.16	0.15	−0.13 to 0.44
Educational level (years)	0.42	0.02	0.39 to 0.46
	Model 3		
IL-6 (pg/ml)	−0.05	0.01	−0.07 to −0.03
Age (years)	−0.16	0.01	−0.19 to −0.14
Sex (Female)	0.24	0.16	−0.06 to 0.55
Educational level (years)	0.41	0.02	0.37 to 0.45
BMI (kg/m^2^)	−0.04	0.02	−0.07 to −0.01
LDL Cholesterol (mg/dl)	0.01	0.01	−0.01 to 0.02
AST (U/L)	0.01	0.01	−0.01 to 0.01
RBC (10^6^ cells/mm^3^)	0.06	0.08	−0.09 to 0.21
Depression (yes)	−0.76	0.22	−1.2 to −0.32
	Model 4		
IL-6 (pg/ml)	−0.05	0.01	−0.07 to −0.03
Age (years)	−0.17	0.01	−0.19 to −0.14
Sex (Female)	0.24	0.16	−0.07 to 0.54
Educational level (years)	0.41	0.02	0.37 to 0.45
BMI (kg/m^2^)	−0.04	0.02	−0.07 to 0.02
LDL Cholesterol (mg/dl)	−0.01	0.01	−0.01 to 0.02
AST (U/L)	−0.01	0.01	−0.01 to 0.01
RBC (10^6^ cells/mm^3^)	0.06	0.08	−0.09 to 0.21
Depression (yes)	−0.77	0.22	−1.21 to −0.33
CRP (mg/dl)	0.02	0.09	−0.16 to 0.2
TNF-α (pg/dl)	0.02	0.02	−0.02 to 0.06

Legend: BMI: Body Mass Index, AST:aspartate aminotransferase, RBC: Red Blood Cells, CRP: *C*-reactive Protein, TNF-α: Tumor Necrosis Factor alpha.

Model 1 revealed that concentration of IL-6 is inversely associated with MMSE score (β = −0.13; 95% CI: −0.14 to −0.12) (Table 3a). The association was still significant in Model 2, after adjustment for age (β = −0.15; 95% CI: −0.17 to −0.13), sex (β = −0.37; 95% CI: −0.62 to −0.11) and educational level (β = 0.29; 95% CI: 0.25 to 0.32). In Model 3, IL-6 levels were significantly associated with MMSE score with adjustment for age (β = −0.15; 95% CI: −0.17 to −0.13), sex (β = −0.34; 95% CI: −0.61 to −0.06), educational level (β = 0.28; 95% CI: 0.25 to 0.32), BMI (β = −0.03; 95% CI: −0.06 to −0.01), depression (β = −0.54; 95% CI: −0.94 to −0.15), LDL Cholesterol, AST and RBC. Finally, in Model 4, the association between IL-6 levels and MMSE score was also significant after adjustment for age (β = −0.15; 95% CI: −0.17 to −0.13), sex (β = −0.35; 95% CI: −0.63 to −0.08), educational level (β = 0.28; 95% CI: 0.25 to 0.32), BMI, LDL Cholesterol, AST, RBC, depression (β = −0.53; 95% CI: −0.93 to −0.14), CRP and TNF-α (β = 0.04; 95% CI: 0.01 to 0.07). The latter regressor (TNF-α) became positively associated after the adjustment for other covariates, hence it seemed more a mathematical artifact than an interpretable result.

Concerning global executive functioning, we found in Model 1 that the concentration of IL-6 was inversely associated with FAB score (β = −0.11; 95% CI: −0.12 to −0.09) (Table 3b). In Model 2, the association was still significant after adjustment for age (β = −0.16; 95% CI: −0.19 to −0.14), sex and educational level (β = 0.42; 95% CI: 0.39 to 0.46). In Model 3, IL-6 levels were significantly associated with FAB score with adjustment for age (β = −0.16; 95% CI: −0.19 to −0.14), sex, educational level (β = 0.41; 95% CI: 0.37 to 0.45), BMI (β = −0.04; 95% CI: −0.07 to −0.01), depression (β = −0.76; 95% CI: −1.2 to −0.32), LDL Cholesterol, AST and RBC. Finally, in Model 4, the association between IL-6 levels and FAB score was also significant after adjustment for age (β = −0.17; 95% CI: −0.19 to −0.14), sex, educational level (β = 0.41; 95% CI: 0.37 to 0.45), BMI, LDL Cholesterol, AST, RBC, depression (β = −0.77; 95% CI: −1.21 to −0.33), CRP and TNF-α.

When MMSE and FAB were used as dependent variables and CRP and TNF-α serum as regressors, we found that TNF-α levels and CRP levels were not significantly associated with MMSE and FAB scores (Table 3c, Table 3dc and 3d).

**Table 3c:** Rank-based Regression model on MMSE as dependent variable and CRP and. TNF-α serum levels as regressors.

	Coefficient	Stand. Err.	CI 95%
	Raw model		
TNF-α (pg/ml)	0.001	0.01	−0.01 to 0.01
	Raw model		
CRP (mg/dl)	0.001	0.01	−0.02 to 0.03

Legend: TNF-α: Tumor Necrosis Factor alpha, CRP: *C*-reactive Protein.

**Table 3d:** Rank-based Regression model on FAB as dependent variable and CRP and TNF-α. Serum levels as regressors.

	Coefficient	Stand. Err.	CI 95%
	Raw model		
TNF-α (pg/ml)	−0.001	0.02	−0.01 to 0.01
	Raw model		
CRP (mg/dl)	0.001	0.01	−0.05 to 0.06

Legend: TNF-α: Tumor Necrosis Factor alpha, CRP: *C*-reactive Protein.

## 4 Discussion

In this population-based study, we investigated the association between serum levels of IL-6, TNF-α, CRP, and cognitive performance in a sample of 1929 South-Italian older adults. We found that higher levels of IL-6 were associated with worse performance on MMSE and FAB tests, even after adjustment for demographic data and cardiovascular risk factors. Prior studies investigating the relationship between serum inflammatory markers and cognition in older adults showed contradictory results [6]. In particular, a recent study in line with our results, showed that increased IL-6 levels were associated with worse executive function and decreased processing speed in older adults, suggesting a higher activation of the innate immune system in subjects with poorer cognitive performance [16]. Moreover, in a cross-sectional multidisciplinary study on aging of the Polish population [17] and in the Health, Aging and Body Composition Study ABC [18], the authors found similar results, showing that higher IL-6 levels were associated with poorer global cognitive performance. From a biomarkers experimental point of view, these results have been confirmed in structural neuroimaging studies since increased IL-6 serum levels were associated with global brain atrophy and white matter lesions at the Magnetic Resonance Imaging in people without cognitive impairment [19,20]. The association between higher IL-6 levels and worse cognitive performance as well as brain atrophy might be explained in part by preclinical studies analyzing human and rat brain tissues which found that IL-6 is mainly found in the hippocampus and, under physiological conditions, higher IL-6 serum levels were associated with reduced grey matter in the left hippocampus [21].

In contrast with our results, the Mayo Study of Aging found no association between IL-6 serum levels and cognitive decline; however, higher levels of IL-6 were cross-sectionally related to higher odds of MCI [22]. Similarly [23], did not observe any significant association between executive function, processing speed, language, memory, global cognitive function IL-6 levels in a community cohort study of 960 European Americans. However, it is noteworthy to mention that the younger age of the study participants in comparison to our cohort that could explain the different results.

The role of IL-6 in cognitive functioning has been studied also in neurodegenerative disorders, where higher serum IL-6 concentrations have been related to an increased risk of dementia and cognitive decline over time [22,24,25]. A recent meta-analysis registered higher levels of peripheral inflammatory markers, including IL-6, in patients with AD and MCI in comparison to healthy controls [26]. Further, increased IL-6 serum levels were found in offspring with a parental history of late onset AD [27]. These findings are in line with several preclinical studies, which showed that IL-6 causes the activation of microglial cells [28] and increases the expression of hyperphosphorylated tau (i.e., [1, 29]. Furthermore, a previous work on experimental rat model of AD showed that the administration of tocilizumab an *anti*-IL-6 receptor antibody, was associated with an improvement in learning and spatial memory functions [30]. These findings further suggest that systemic inflammation might play an important role in AD pathology [28] and cognitive decline in aging.

Concerning *C*-reactive protein, in our study we did not find any significant association between cognitive impairment and serum levels of CRP. This is inconsistent with previous studies which described higher CRP levels related to impaired cognitive performance in older adults [31] and to increased risk 25 years later of dementia [32]. However, other works failed to show any significant association between CRP and cognitive function [33,34] as well as no association between CRP and cognitive decline at 3-year follow-up was found in older adults [35,36]. These contrasting results might be explained by the different ethnic and demographic characteristics of the individuals enrolled in the study.

As well as for CRP, we did not find a significant association between cognitive impairment and TNF-α levels**.** This is in line with previous studies that found no association between TNF-α and cognitive function in older adults enrolled in population-based studies [18,22].

To the best of our knowledge, no studies focused on the sex-related differences when investigating the association between serum cytokines and cognitive functions in older adults. It is well known that several factors contribute to the sex-specific differences in the immune response such as genetic factors, sex hormones as well as gender-specific behaviors and exposures [37]. Overall, it has been reported that the serum levels of pro-inflammatory markers such as CRP, IL-6, TNF-α and IL-10 increase with aging [[38], [39], [40]]. When investigating sex-related differences in the IL-6 and CRP serum concentration Puzianowska-Kuźnicka and colleagues [41] found higher levels of IL-6 in males compared to females, but no differences in CRP concentrations. In our study, we considered sex as a potential confounder in the statistical analysis, however, further studies are needed in order to better explore the role of sex differences in the association between serum cytokines and cognition in older adults.

Our study has many strengths. First, we collected data in a population-based study with a large cohort of individuals, which may be representative of the Southern Italian population. The fact that the study population was community-based and not clinic-based is likely a better reflection of inflammatory markers level in the general population. To our knowledge, it is the biggest community-dwelling study investigating neuroinflammatory markers and cognition in Southern Italians. So far, the majority of population-based studies investigating the relationship between inflammation and cognition included subjects with high educational levels [16,22]. The present study enrolled individuals with low educational and socioeconomic status, in a geographically defined population of Southern Italy. The study participants are older adults with low educational status and who worked as farmers or in small companies [42]. It has been reported that education level and socio-economic disparities might play a role in inflammation, by increasing exposure to unhealthy behaviors and psychological stresses [5,6]. However, study populations with low socioeconomic status are usually under-represented in health research [43]. Thus, our work extends previous evidence by studying the relationship between inflammation and cognition in a rural population with low educational grades, considering demographic data and cardiovascular risk factors as potential confounders in the analysis. Limitations should also be acknowledged. The study was conducted in non-patient samples and no data about morbidities and ongoing therapies were considered, so we cannot draw a conclusion on the causal relationship between cognition and inflammation in specific disease conditions. Further, the cross-sectional design represents a substantial limitation because it does not allow us to make causal inferences between inflammatory markers and cognitive decline over time.

## 5 Conclusions

In conclusion, our study shows that higher IL-6 serum levels are associated with global cognitive impairment and worse executive functioning in a wide sample of older adults in South Italy, suggesting that neuroinflammation mediated by IL-6 plays a role in cognitive decline. Longitudinal studies are needed in order to assess whether IL-6 levels are suitable as a diagnostic tool in order to identify individuals at high risk for dementia. Moreover, preclinical studies and clinical trials are required to investigate whether *anti*-IL-6 drugs, such as tocilizumab, are able to improve cognitive function in normal aging and in dementia.

## Author contribution statement

Chiara Griseta: Performed the experiments; Analyzed and interpreted the data; wrote the paper.

Petronilla Battista, Isabella Colonna: Conceived and designed the experiments; Analyzed and interpreted the data; Wrote the paper.

Fabio Castellana, Rodolfo Sardone: Conceived and designed the experiments; Analyzed and interpreted the data.

This work was partially funded by the Ricerca Corrente funding scheme of the 10.13039/501100003196Italian Ministry of Health.

## Funding statement

This work was partially funded by the Ricerca Corrente funding scheme of the Italian Ministry of Health.

## Data availability statement

Data will be made available on request.

## Declaration of interest's statement

The authors declare that they have no known competing financial interests or personal relationships that could have appeared to influence the work reported in this paper.
